# Application of DNA barcodes and spatial analysis in conservation genetics and modeling of Iranian *Salicornia* genetic resources

**DOI:** 10.1371/journal.pone.0241162

**Published:** 2021-04-23

**Authors:** Mehrshad Zeinalabedini, Nayer Azam Khoshkholgh Sima, Mohammad Reza Ghaffari, Ali Ebadi, Maryam Farsi

**Affiliations:** 1 Department of Systems and Synthetic biology, Agricultural Biotechnology Research Institute of Iran, Agricultural Research, Education and Extension Organization (AREEO), Karaj, Iran; 2 Department of Molecular Plant Physiology, Agricultural Biotechnology Research Institute of Iran, Agricultural Research, Education and Extension Organization (AREEO), Karaj, Iran; Universita degli Studi di Milano-Bicocca, ITALY

## Abstract

Iran is one of the origins of some *Salicornia* species. Nevertheless, comprehensive research has not been conducted on genetic potential, distribution, selection of populations, and the economic utilization of *Salicornia* in Iran. In the current study, *Salicornia* was collected based on the previous data available for 26 different geographical locations of provinces in Iran. We examined Salicornia plants’ universality DNA barcodes, including rbcL, matK, trnH-psbA, and ITS, and their species identification abilities and identified six species groups. Subsequently, accurate modeling of distributed areas was provided with MAXENT and highlighted the valuable information on the diversity of specific geographical regions, conservation status of existing species, prioritization of conservation areas, and selection of Agro-Ecological areas. Together, this type of integrative study will provide useful information for managing and utilizing *Salicornia* genetic resources in Iran.

## Introduction

The world’s population is 7 billion in 2018 and is expected to rise by 35% and reach 9 billion in 2050. Food and Agriculture Organization of United Nations (FAO) estimates crop production must increase by at least 60% in the future to provide food security for the world’s growing population [1,2]. However, the use of genotypes with high-yield and more resistant to biotic and abiotic stress has gradually replaced indigenous varieties and local genotypes, endangering access and exploitation of resources and food production for the future [3]. Thus, low genetic diversity may reduce the opportunity to identify and use new sources responding to future challenges, including new pests and pathogens, as well as climate changes [4]. Today, soil salinity has increased due to unconventional cultivation and irrigation. Moreover, the level of arable land increased dramatically from 8 million to more than 220 million hectares, of which about 45 million hectares are salt-affected to varying degrees [5,6].

Halophytes, which include more than 600 taxa of the various genus and species [7], complete their life cycle under high salt concentration (at least 200 mM NaCl) [8,9]. *Salicornia* is a succulent halophyte, which grows naturally on mangrove swamps as well as seashores. It has also been evaluated as a vegetable, forage, and oilseed crop in the agronomic field trials [10]. There is a morphological variation across the genus distribution range of *Salicornia* sp. Some economically feasible applications have been suggested for *Salicornia* species. They also have suitable biomass for human and domestics consumption [11,12]. Furthermore, *Salicornia* seed contains 26%–33% oil, similar to safflower oil, along with 30%–33% protein [13].

Members of the Salicornioideae are promising candidates for saline agriculture [14]. For instance, *Salicornia bigelovii* Torr., a true halophyte, is a potential new crop due to the oil content, fresh vegetable, forage, and its seeds in coastal and saline lands. In recent years, Salicornia has been cultivated for various purposes around the world, from the production of Salicornia oil by a Saudi company to its cultivation by the UAE Institute of Science and Technology to produce aircraft biofuels [14]. The highest saline soils in Asia are China, India, Pakistan, and Iran [15]. Iran is located in arid and semi-arid geographical areas rich in the distribution of naturally occurring materials such as halite (NaCl) and gypsum (CaSO4 covering 34% of the regions of the country, especially in the central, south, and plain of Khuzestan in varying degrees of salinity [7]. Furthermore, about 20%-50% of the arable land is also affected by salinity [8,9].

Salicornia sp. is widespread across Iran, mainly in central, western, and northern parts. Thus, native *Salicornia* species selection might be a viable agricultural development strategy against Iran’s climate change. Pilot cultivation of Salicornia in different regions of Iran and study of its forage quality in collaboration with Animal Science Research Institute of IRAN (ASRI) showed that this plant could supply up to 21% of the volume of forage for feeding of light livestock such as sheep (Unpublished).

The number of *Salicornia* species is between 25 to 30 species worldwide [16]. The taxonomy classification of *Salicornia* is very complicated as there is no general published morphological descriptor for all accepted species. Previously, some morphological characteristics like growth form, inflorescence, branching of main and lateral stems, lateral flowers and their conditions relative to the main flowers, fruit formation, flowering, and fruiting characteristics as well as dry biomass were used to identify some species of *Salicornia* with high seed and biomass yield [17–19]. However, a low number of morphological characteristics, phenotypic flexibility, breeding, and hybridization systems are amongst the factors that make *Salicornia* challenging to distinguish precisely in an area [20]. Thus, the commercial importance and lack of description make *Salicornia* a priority for the circumscription of species, subtypes, ecotypes, and natural hybridization to develop it as a new crop [21]. Currently, few studies were conducted on taxonomic and phylogenetic analysis of the *Salicornia* genus. Teege et al. [21] studied the genetic relationships of intra and inter-variation of the two taxa of *Salicornia prompumbens* and *Salicornia stricta* species using AFLP markers [17]. Further, the phylogenetic relationships between eight taxa belonging to the *Salicornia* genus were examined using the ITS markers [21]. Likewise, the phylogenetic and geographic relationship in different germplasms of *Salicornia* was evaluated using six EST markers [21].

Moreover, the genetic diversity of some populations of *Salicornia* was investigated using RAPD markers [22]. All obtained results showed that molecular markers have some limitations/disadvantages inaccurate taxonomic classifications. DNA sequencing technology has recently facilitated identifying identical genotypes with different morphotypes or different genotypes with the same morphotypes [23]. DNA barcoding, which is a new and efficient tool based on the conserved DNA regions of organisms, has provided an efficient and precise method for species-level identifications [24]. DNA barcodes were developed by Consortium for the Barcode of Life (CBOL) as a global standard for the identification of biological species. To date, no comprehensive study has been performed on the utility of universal DNA barcodes for all proposed gene regions (either alone or in combination) in *Salicornia*. The overall aim of our study was to employ universality of three coding plastid regions (*rbcL*, *matK* and *ycf*), one non-coding plastid intergenic spacer regions (*trnH-psbA*), and the internal transcribed spacer of nuclear-encoded ribosomal DNA (ITS2) to identify the Salicornia species using molecular data and ecological niche. An integrated model was then developed to assess Salicornia distribution and best habitat based on climate factors and estimate critical parameters in land management and biodiversity conservation. We assume that our results will open up a new window for the future development of *Salicornia* farms.

## Materials and methods

### Sample collection

**Fig 1:**
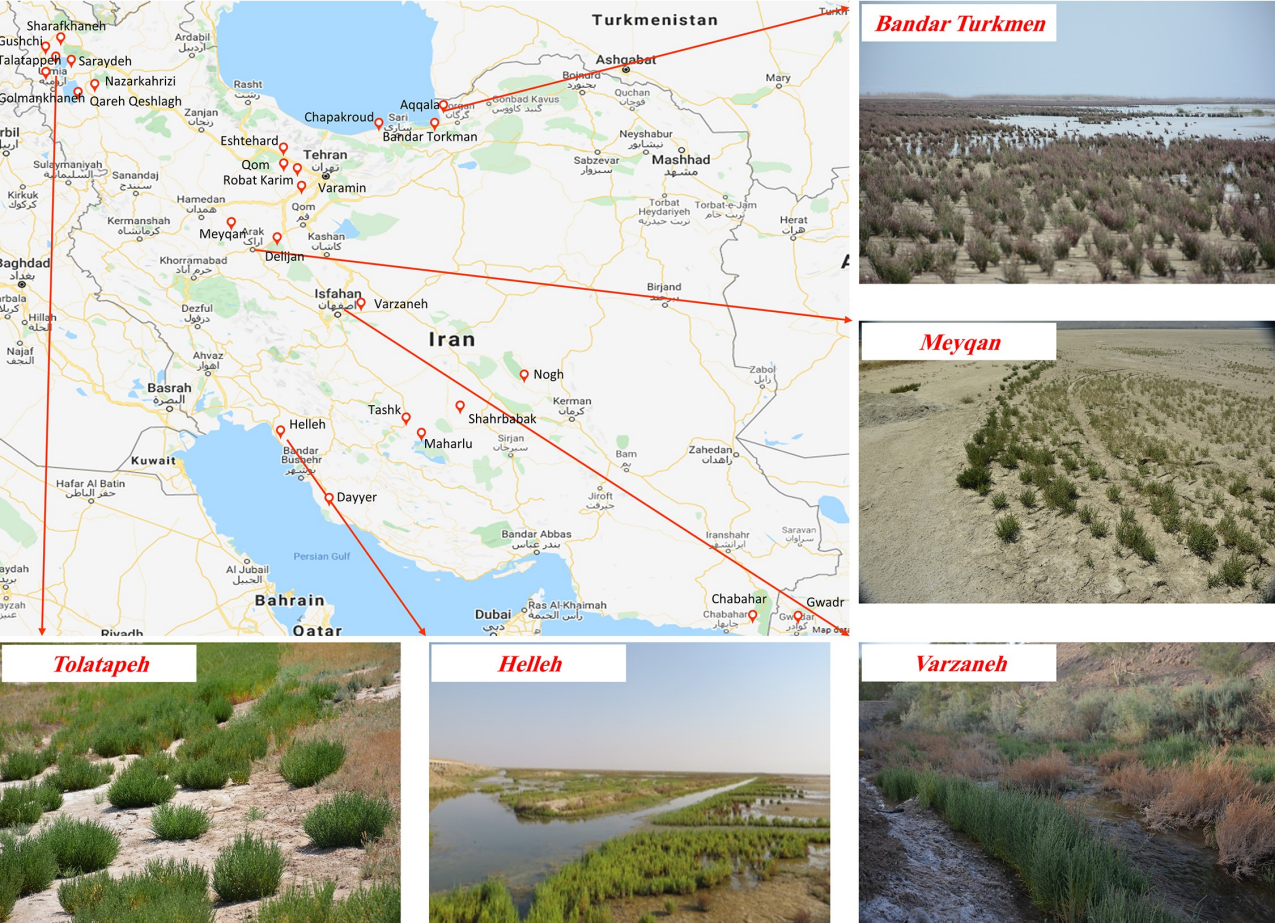
Distribution map of *Salicornia* germplasms in Iran. The gateway to astronaut photography of earth was used to create the map (https://eol.jsc.nasa.gov/).

The previously collected records by the Agricultural Biotechnology Research Institute of Iran (ABRII) were used to identify distribution localities for sampling [25]. Field surveys were then done to collect samples from populations throughout entire natural distribution of *Salicornia* sp in Iran. Twenty-six different geographical locations were included in this research (Fig 1). A total of 300 samples were collected from different geographical areas ranging from 2 to 5 samples per location (Table 1). A minimum of 91 required samples was selected for sequencing. At least 3–5 samples were successfully sequenced and analyzed.

**Table 1:** Geographic information of *Salicornia* populations that were collected in this study.

No.	Province	Collection Site	Longitude	Latitude
1	Alborz	Jafar Abad	50^ᶱ^ 49ʹ 26.511ʺ	35^ᶱ^ 42ʹ 38.401 ʺ
2	Alborz	Eshtehard	50^ᶱ^ 30ʹ 20.195ʺ	35^ᶱ^ 44ʹ 45.052 ʺ
3	Markazi	Meyghan	49^ᶱ^ 48ʹ 56.527ʺ	34^ᶱ^ 10ʹ 12.262 ʺ
4	Markazi	Delijan	50^ᶱ^ 49ʹ 58.401ʺ	33^ᶱ^ 48ʹ 38.401 ʺ
5	Isfahan	Varzaneh	52^ᶱ^ 39ʹ 53.002ʺ	32^ᶱ^ 25ʹ 22.763 ʺ
6	Mazandaran	Chapak roud	52^ᶱ^ 51ʹ 48.51ʺ	36^ᶱ^ 43ʹ 9.602 ʺ
7	West-Azerbayjan	Gulmanxana	45^ᶱ^ 16ʹ 17.644ʺ	37^ᶱ^ 35ʹ 13.912 ʺ
8	West-Azerbayjan	Talatappe	45^ᶱ^ 13ʹ 27.699ʺ	37^ᶱ^ 44ʹ 54.563 ʺ
9	Qom	Qom highway	51^ᶱ^ 10ʹ 43.625ʺ	35^ᶱ^ 19ʹ 18.318 ʺ
10	Sistan & Blaochestan	Chabahar	60^ᶱ^ 40ʹ 20.423ʺ	25^ᶱ^ 17ʹ 21.001 ʺ
11	Sistan & Blaochestan	Gwadr	62^ᶱ^ 22ʹ 29.560ʺ	25^ᶱ^ 20ʹ 6.936 ʺ
12	Tehran	Robat Karim	50^ᶱ^ 57ʹ 44.412ʺ	35^ᶱ^ 26ʹ 37.730 ʺ
13	Tehran	Varamin	51^ᶱ^ 17ʹ 40.124ʺ	34^ᶱ^ 56ʹ 53.952 ʺ
14	Golestan	Bandar Torkamen	54^ᶱ^ 3ʹ 0.072ʺ	36^ᶱ^ 53ʹ 18.160 ʺ
15	Golestan	Aq Qala	54^ᶱ^ 22ʹ 46.480ʺ	37^ᶱ^ 5ʹ 58.844 ʺ
16	Boshehr	Dayyer	51^ᶱ^ 58ʹ 31.501ʺ	27^ᶱ^ 50ʹ 31.642 ʺ
17	Boshehr	Helleh	50^ᶱ^ 49ʹ 52.812ʺ	29^ᶱ^ 11ʹ 22.106 ʺ
18	East-Azerbayjan	Nazarkahrizi	46^ᶱ^ 54ʹ 52.339ʺ	37^ᶱ^ 21ʹ 34.416 ʺ
19	East-Azerbayjan	Qushchi	45^ᶱ^ 4ʹ 39.946ʺ	37^ᶱ^ 59ʹ 40.941 ʺ
20	East-Azerbayjan	Sharafkhaneh	45^ᶱ^ 28ʹ 33.041ʺ	38^ᶱ^ 10ʹ 2.236 ʺ
21	East-Azerbayjan	SarayDeh	45^ᶱ^ 38ʹ 48.551ʺ	37^ᶱ^ 52ʹ 47.262 ʺ
22	East-Azerbayjan	Qareh Qeshlaq	45^ᶱ^ 58ʹ 6.110ʺ	37^ᶱ^ 13ʹ 46.699 ʺ
23	Fars	Tashk	53^ᶱ^ 35ʹ 41.686ʺ	29^ᶱ^ 49ʹ 15.232 ʺ
24	Fars	Maharlu	53^ᶱ^ 45ʹ 50.043ʺ	29^ᶱ^ 31ʹ 45.922 ʺ
25	Kerman	Nogh	56^ᶱ^ 0ʹ 0219ʺ	30^ᶱ^ 39ʹ 17.028 ʺ
26	Kerman	Shahrbabak	54^ᶱ^ 39ʹ 56.807ʺ	30^ᶱ^ 6ʹ 8.463 ʺ

### Molecular analysis

#### DNA extraction and barcoding

DNA extraction was carried out using the Core Bio DNA extraction kit (Core Bio, South Korea). The quality and quantity of DNA were determined using 0.8% agarose gel and NanoDrop ND-1000 spectrophotometer (Nanodrop Technologies, Wilmington, DE, USA), respectively. DNA barcoding was carried out using core-barcode consisting of two plastid coding regions, (*rbcL*+*matK*) and supplemented with one non-coding intergenic spacer (*trnH-psbA*) from the chloroplast genome, one internal transcribed spacer (*ITS2*) from the nuclear genome, and one plastid coding region (*ycf*) [24,26] (see S1 Table). For all markers, 50 μl reactions containing 1 U Taq DNA polymerase (Invitrogen or Promega), 2.5 μl Taq DNA polymerase buffer (10X), 1.5 μl MgCl_2_ (25 mM), 0.5 μl dNTPs (10 mM), 0.35 μl primers (20 pmol), 1.0 μl template DNA and 18.85 μl dH_2_O were carried out in a Veriti ABI thermal cycler (ABI Inc, USA). Then, the PCR products were purified using PureLINK PCR product purification kit (Thermo Fisher Scientific). The purified PCR product was then directly sequenced in both directions to minimize PCR artifacts, ambiguities, and base-calling errors on an automated ABI Prism 3730 XL Genetic Analyzer machine using ABI BigDye v3.1 Terminator Sequencing chemistry (Macrogen Inc, South Korea). In this study, the GC content threshold above 65% was considered high GC content. GC content is a good predictor of PCR success and can significantly impact the PCR success rate and sequencing error rate. It is also an indicator of binding ability and specificity (primer binding and specificity) to optimize the DNA barcoding process. Therefore, the sequenced samples with above 65% GC content were cloned and then sequenced.

The sequencing results were then sorted and trimmed using FinchTV software (https://digitalworldbiology.com/FinchTV) and analyzed by protein BLAST (BLASTP), nucleotide (BLAST) BLASTN [27], and LALIGN software (https://embnet.vital-it.ch/software/LALIGN_form.html). Finally, DNA barcode data compared to GenBank or otherwise publicly available in the BOLD database based on different integration strategies (coding, non-coding, plastid, and total combination) using a similarity-based method. The sequence similarity-based method was briefly utilized for species identification, using simple and optimized BLAST methods for different marker integration strategies (single, coding, non-coding, plastid, and total combination). First, sequences were queried using megablast using Geneious software (Biomatters) and NCBI (national center for biotechnology information) BLASTn against the nucleotide database. A simple method was then considered the top 10 hits, and the top 100 records were used to put extra weight on the identity value using "max score*(query cover/identity)" formula using the optimized BLAST method. Finally, taxonomic identifications were allocated based on the combination of the identity score (High identity: X≥95%; Medium identity: 90% ≤ X ≤ 95%; Low identity: X ≤ 90%) and the number of species within 1% deviation of the calculated similarity score (S1 Fig). Plant Sequences database (BOLD systems) and ITS2 database (V5) were also used as a validation tool for the core barcodes (matK, rbcL) and ITS2 [28,29]. Phylogenetic tree was obtained by MEGA X version 10.2.2 (https://www.megasoftware.net/) by analyzing all the sequence data.

### Analysis of genetic diversity

Analysis of molecular variance (AMOVA) was carried out using Arlequin version 3.5 [30]. The significance level of *F*_*st*_ statistics was also performed using the software by a nonparametric permutation procedure with 1023 randomizations. Statistical calculations and graphics for *F*_*st*_ were conducted using Arlequin and R version 3.6.1 (cran.r-project.org) [31]. Further, the online version of Automatic Barcode Gap Discovery (ABGD) (https://bioinfo.mnhn.fr/abi/public/abgd/abgdweb.html) [32] was used to generate the genetic distance histograms and ranked distance based on K2P distance. The ABGD detects a gap. Then, when a gap exists in the distribution of divergence corresponded to differences between intra-specific and inter-specific distances, the method will work well for species delimitation. The following setting was used to perform the analysis: relative gap width X  =  1.5, K2P distance, and intraspecific divergence (P) values range from 0.001 to 0.100. Further parameter values were employed based on the software defaults. The analyses were performed at the web server. In the ABGD system, specimens identified as one group could be considered as one species [32].

### Species distribution modeling

The modeling program MAXENT v.3.3.3 was used in the default settings with a random test percentage of 25% of the input localities set aside for modeling Iranian *Salicornia* sp. potential distribution. The maximum entropy algorithm calculates the ecological niche modeling, determines the potential natural distribution of areas, and limits climatic factors using presence-only sampling data and environmental data layers [33].

Briefly, nineteen environmental variables (BIOCLIM) were provided by the web-based platform, WorldClim database (www.WorldClim.org) version 1.3, October 2004 [34], which includes climate databases from different sources. Environmental data were then further added from the FAO Map on Global Ecological Zones to build up the potential natural distribution model [35]. Then, the climate data information downloaded from www.WorldClim.org, was converted into a ESRI ASCII GIS (.asc) file by DIVA-GIS for the software. The data were first filtered, and outliers were then detected in DIVA-GIS (www.diva-gis.org) [36]. Then, all occurrence records and extreme values (minimum of 3 out of 19 bioclimatic variables examined) were rechecked for the inconsistency of their coordinates with administrative area level 1 and their climatic parameters based on the Reverse Jackknife method [37].

### Modeling validation

The AUC of the receiver operating characteristic (ROC) was used to assess the constructed model [38]. The AUC value statistics as an independent threshold was ranged from 0.5 to 1, where a value below 0.5 was interpreted as a random prediction; 0.5–0.7 showed poor model performance; 0.7–0.9 showed moderate model performance, and a value above 0.9 was considered to have ’good’ discrimination abilities [39,40].

## Results and discussion

### Universality and evaluation of DNA barcodes for Salicornia sp

**Fig 2:**
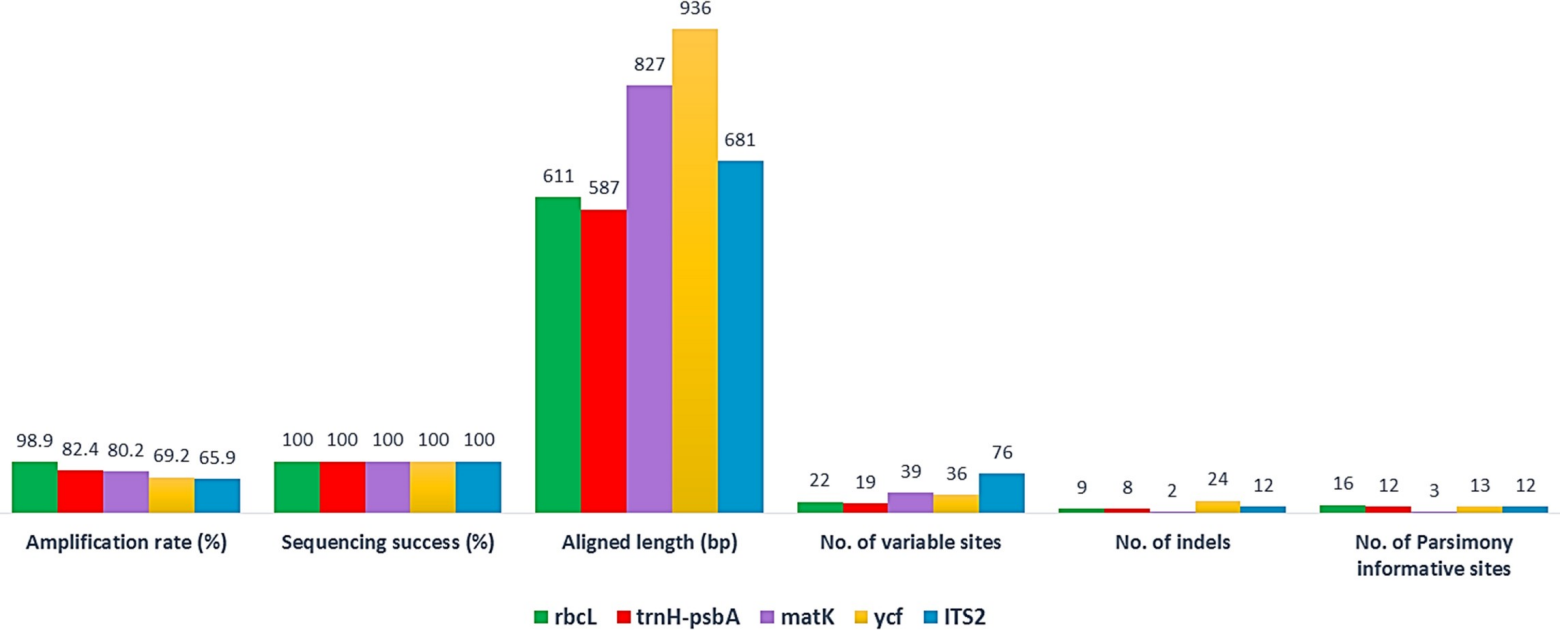
The success rates of PCR amplification and sequencing of the five-barcode fragment and 26 different geographical locations.

The success rates of PCR amplification and sequencing of the five barcode markers and their phylogenetic tree using single barcodes are shown in Fig 2 and S1 File. The three genomic and plastid DNA regions, including *ITS2*, *trnH*-*psbA* spacers and *ycf* genes and a combination of *matK*-*rbcL* were used as acceptable standard barcodes. Among the markers tested, *rbcL* had the highest amplification and recovery rates (98.90%), followed by *trnH*-*psbA* (82.42%), *matK* (80.21%), *ycf* (69.23%) and the rate for *ITS2* was the lowest (65.93%). A low percentage amplification rate and recovery for *ITS2* were due to the lack of suitable public primer, the incongruence of multiple copies, and or technical problems. Although the amplification success rate of *ITS2* is low, it has been commonly used in studies of systematic and evolutionary botany. For example, *ITS2* to be the most suitable region of more than 6,600 plant samples belonging to 4,800 species from 753 distinct genera tested, with 92.7 percent discrimination ability at the species level [41]. The sequence alignment method comprised 60 ITS2 sequences, 73 *matK* sequences, 90 sequences of *rbcL*, 75 sequences of *trnH-psbA*, and 63 sequences of *ycf*.

**Fig 3:**
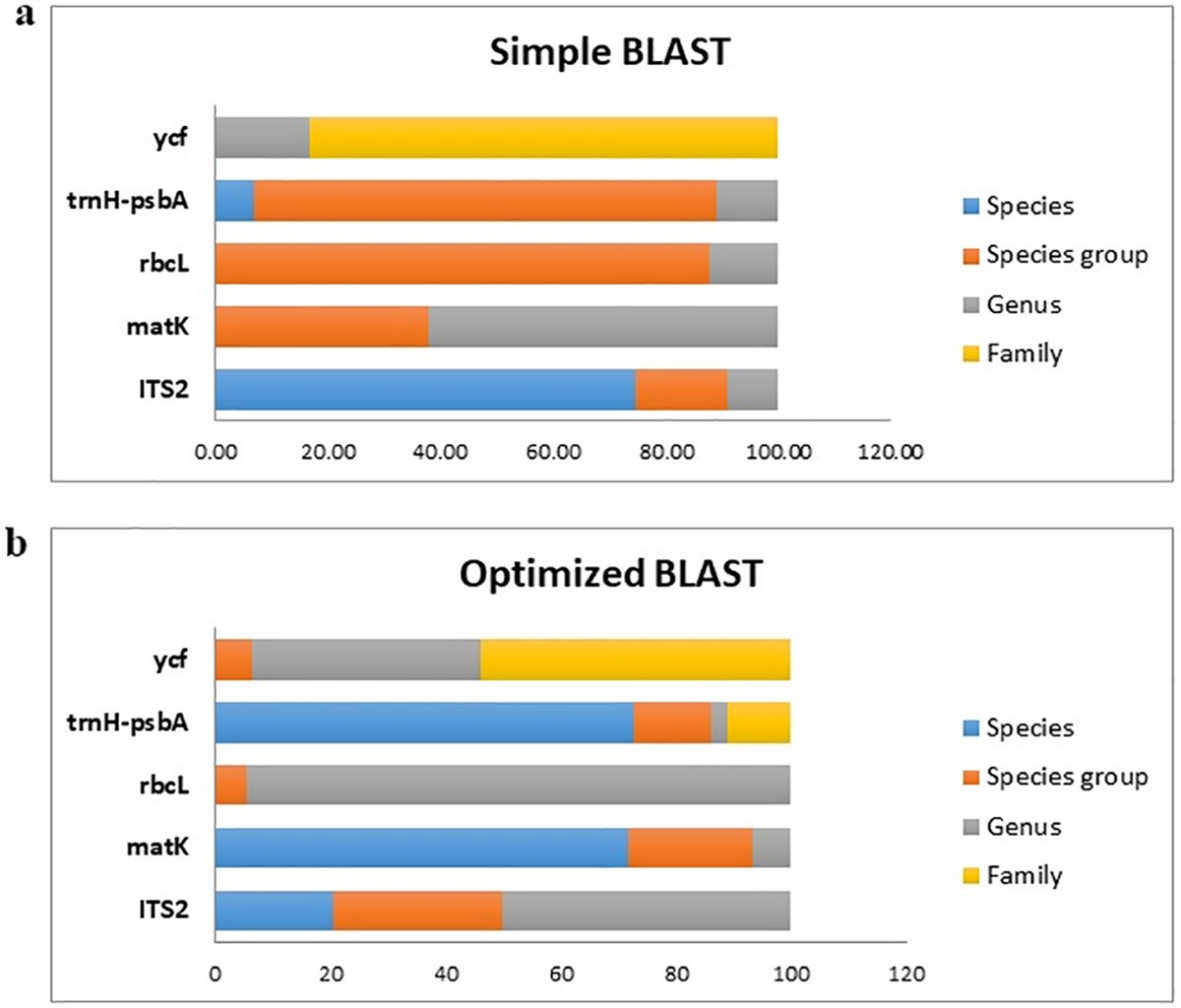
Comparison of DNA barcoding efficiency using selected regions; a) simple and b) optimized BLAST based on NCBI and ITS database.

**Furthermore,** simple and optimized BLAST were carried for each of these sequences based on NCBI and ITS database. The efficiency of every single marker, as well as their combination for taxonomic identification, is presented in Fig 3. The highest level of identification using the single marker and simple BLAST at the species level, species group, genus, and family groups were obtained for *ITS2* (74.55%), *rbcL* (98.78%), *matK* (62.19%) and *ycf* (83.08%) respectively while the discrimination efficiency using optimized BLAST was 70% (*matK* and *trnH*-*psbA*), 29.63% (ITS2), 94.44% (*rbcL*) and 53.96% (*ycf*) respectively (Fig 3). Likewise, data integration showed a higher identification at the level of the species group [42]. This group included *S*. *persica*, *S*. *europea*, *S*. *patula*, *S*. *brachiate*, *S*. *herbacea* and *S*. *maritime* suggesting species such as *S*. *europea*, *S*. *patula* and *S*. *herbacea* are similar or synonymous (The Plant List (2013). Version 1.1. Published on the Internet; http://www.theplantlist.org/ (accessed 1st January)).

### Specific barcode for Salicornia sp

**Fig 4:**
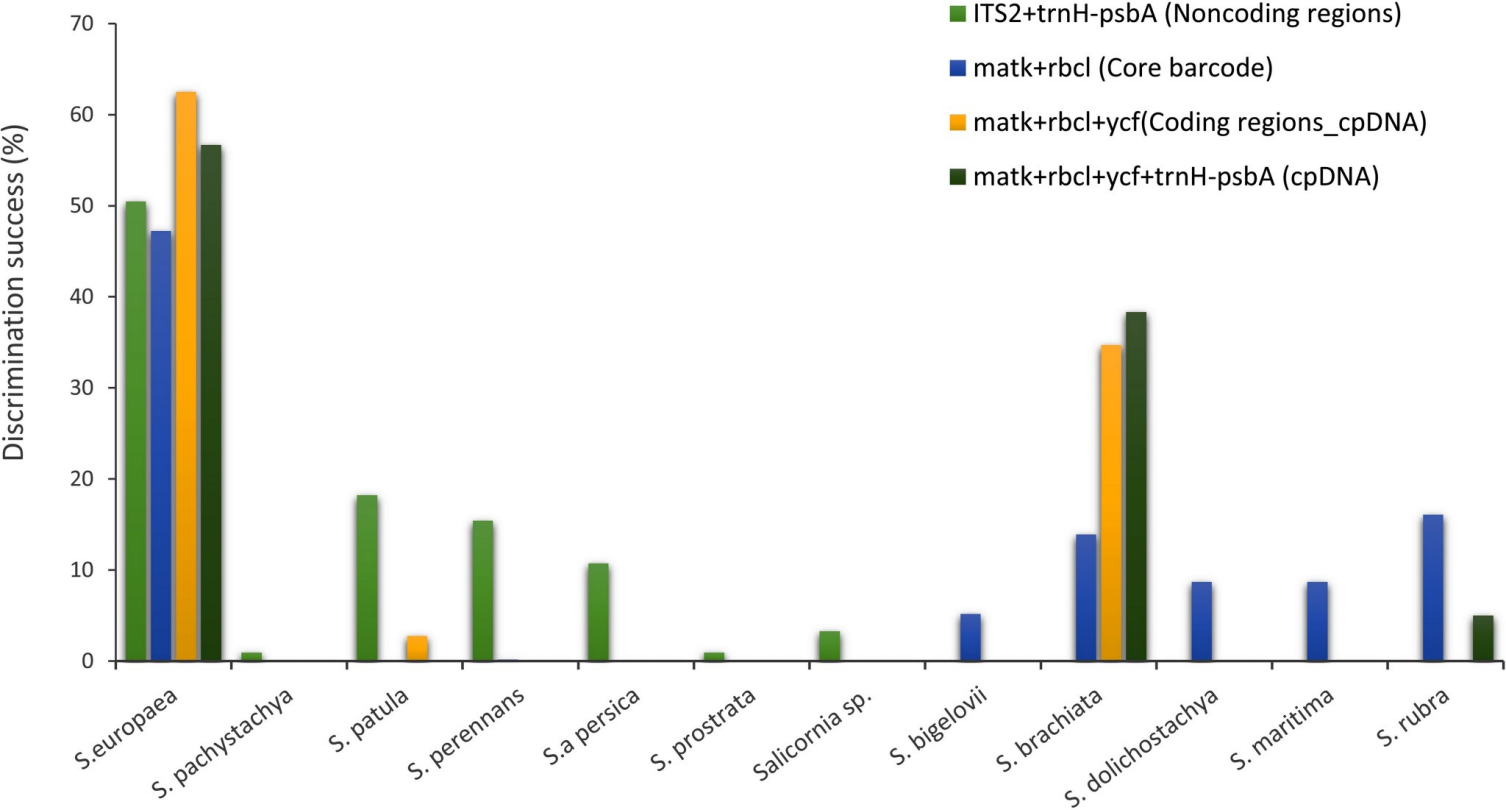
Performances of *matK*, *rbcL*, *trnH*-*psb*A, *ITS2* and *ycf* in resolving *Salicornia* species as different non-coding, coding, core and cpDNA combinations.

Automatic barcode gap discovery (ABGD) analysis, which assigns the sequences into potential species based on the barcode gap when the divergence within the same species is smaller than that among organisms from different species, detected a barcoding gap between the intraspecific and interspecific distance of barcode markers. Based on the distance-based approach as implemented in the software ABGD, different groups as candidate species were produced for rbcL, matk, ycf, psbA gene sequences. We analyzed K2P for distinct barcode gaps. No barcode gap is visible, including all samples and markers studied. For example, the study of matK identified the presence of two hypothetical species in all recursive partitions with prior intraspecific genetic divergence values between 0.46% and 10%, a result we considered four species with intraspecific divergence values below 0.28%. A barcoding gap between 3% and 16% of pairwise distances was also observed by the ABGD procedure. By the study of other markers, the two species hypothesis was also obtained. It should be noted that the ABGD algorithm yielded the same results when applied in the three implemented models (JC, K2P, and Simple Distance). Histograms of sequence divergence values and ranked distances among barcode sequences in *Salicornia* complex are shown in S2 Fig. The highest discrimination success in all combinations obtained for the *S*. *europea* species followed by *S*. *brachiata* (Fig 4). Further, the low success rate of markers might be related to the spreading and distribution of *Salicornia* germplasm species in Iran. This index is inverted with the amount of intra and inter-species gene flow. According to this theory, populations with a low distribution index are geographically or physiologically related. The low distribution index may cause the variants of neutral mutations to spread slowly throughout the entire population. Thus, the required time for lineage sorting for each gene locus increase by the natural genetic flow. In this case, the identification of specific barcodes is difficult for each species [43].

Another consequence is that an increase in the penetration coefficient of a species in the adjacent species is caused by genetic flow, affecting the DNA barcoding method’s efficiency rate. In this study, the barriers and geographic distances, self-pollination, and cleistogamy reduced the gene flow rate among the plausible species of *Salicornia* populations. Later, the Iranian *Salicornia* germplasm has been studied with respect to the different nuclear and plastid sites with different heritability patterns in each group. As previously reported, the inheritance of plastid DNA is maternal in most angiosperms. Thus plastid haplotypes disperse through seeds [44]. In contrast, nuclear variants are distributed by seed and pollen and exhibited by biparental inheritance. The expected distribution range of pollen is higher relative to seed. Thus the genetic differentiation of the populations should be higher through plastid markers [45–47].

### DNA barcodes variation survey on Salicornia population

**Fig 5:**
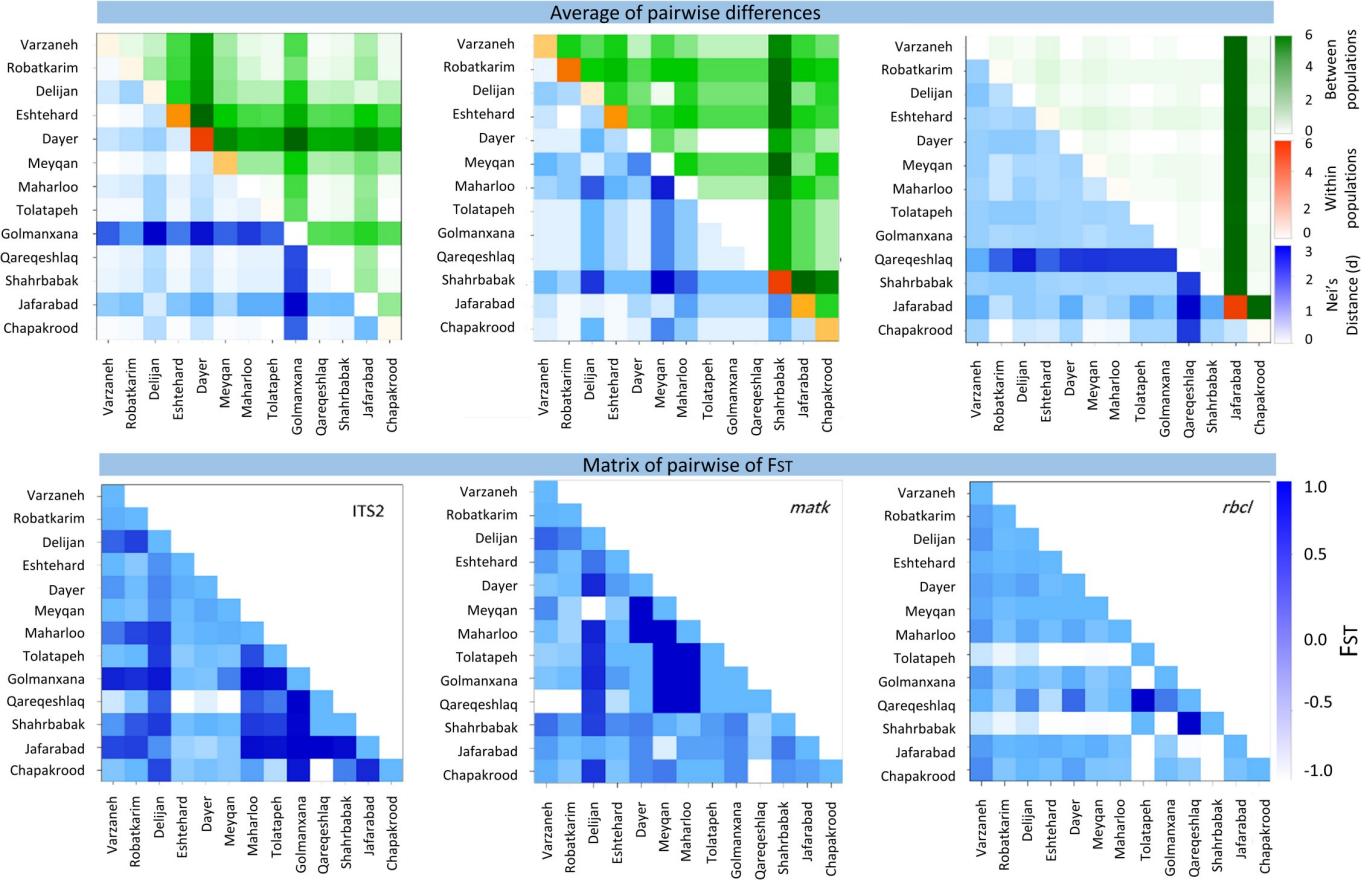
Inter and intra molecular variance analysis and *F*_*st*_ index in *Salicornia* germplasm in Iran, based on plastid and nuclear markers.

Here, molecular variance analysis (AMOVA) and *F*_*st*_ index for core-barcode plastid (*matK*, *rbcL*) and nuclear marker (*ITS2*) showed that molecular variance within populations of the same species is greater than between populations indicating a higher genetic diversity (Fig 5). The largest difference between and within populations was observed in Dayer and Shahrbabbak genotypes based on *ITS2* and matK markers, respectively. However, the rbcL fragment was not suitable for differentiation between populations. The highest Nei’s distance was obtained in Golmanxana and Qaregheshlaq genotypes based on *ITS2* and rbcL markers. Moreover, the highest Fst index was observed in the Golmanxana genotype for the *ITS2* marker, suggesting a low genetic flow rate compared to other genotypes. A higher Fst index conserves the allelic diversity needed for the conservation and exploitation of genetic resources. Further, the distribution of seed and pollen varies among populations, and in most cases, the genetic differentiation between populations depends on the geographical barriers and distance [48].

### Mapping species diversity

**Fig 6:**
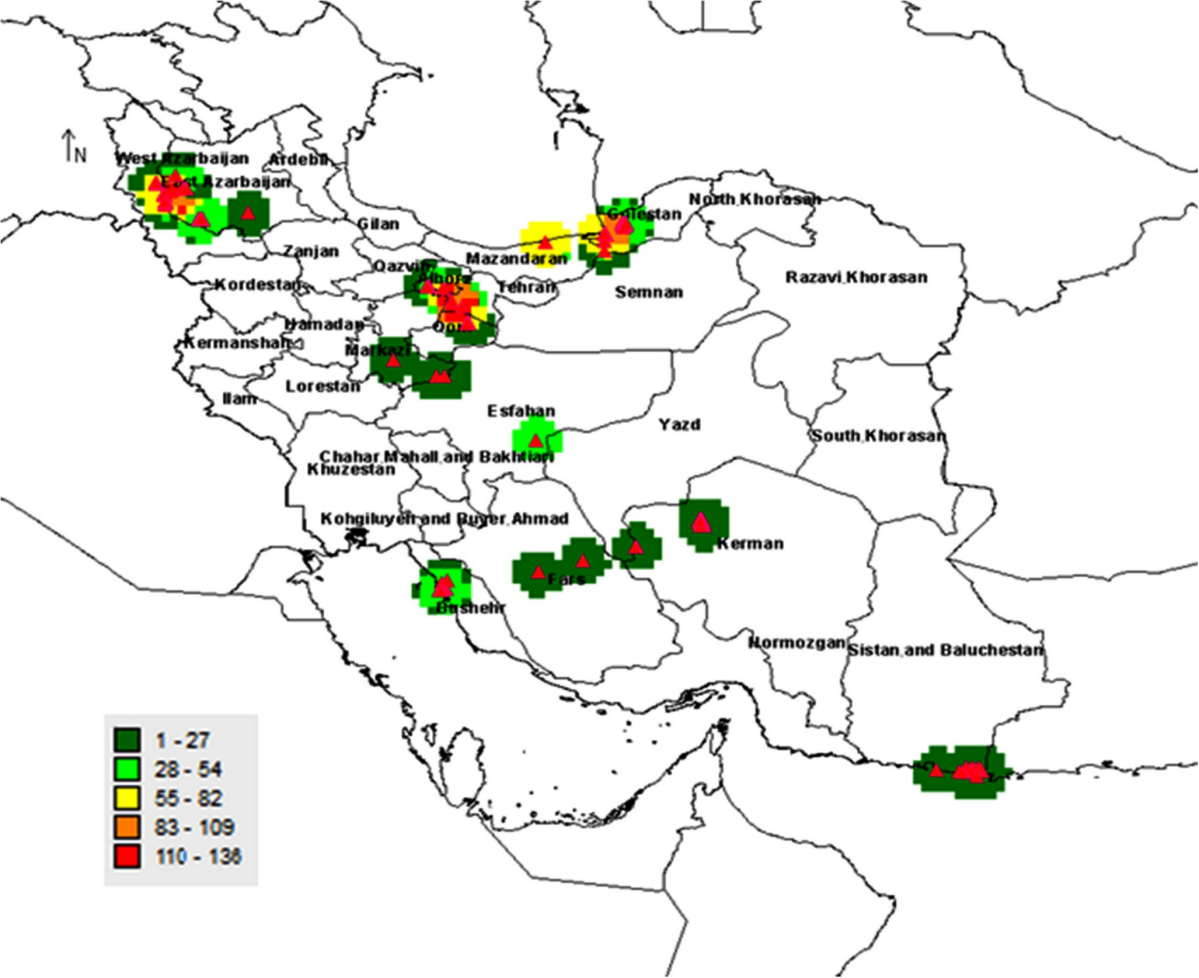
Distribution of the *Salicornia* populations in Iran based on the sampling areas. The regions with the highest and lowest samples are depicted in red and dark green, respectively. The map was created using free software DIVA-GIS Version 7.5 (www.diva-gis.org).

The spatial analysis helps in better understanding and more accurate identification of biodiversity and developing strategies for identifying, managing, and exploiting genetic resources [49]. Outputs then provide vital information for prioritizing of protection and the Agro-Ecological Areas [50] finding. These findings may suggest that the new species can be grown in an environment of outside of its endemic region if additional water or soil nutrients are available. It should be noted that combining spatial results with other data is very useful in the management and exploitation of genetic reserves more efficiently [51]. The basis of spatial analysis in biodiversity is observed data from the sampled collection areas. In our study, the geographical data included identification codes, taxonomic names, geographic characteristics, and sampling locations (Fig 6).

**Fig 7:**
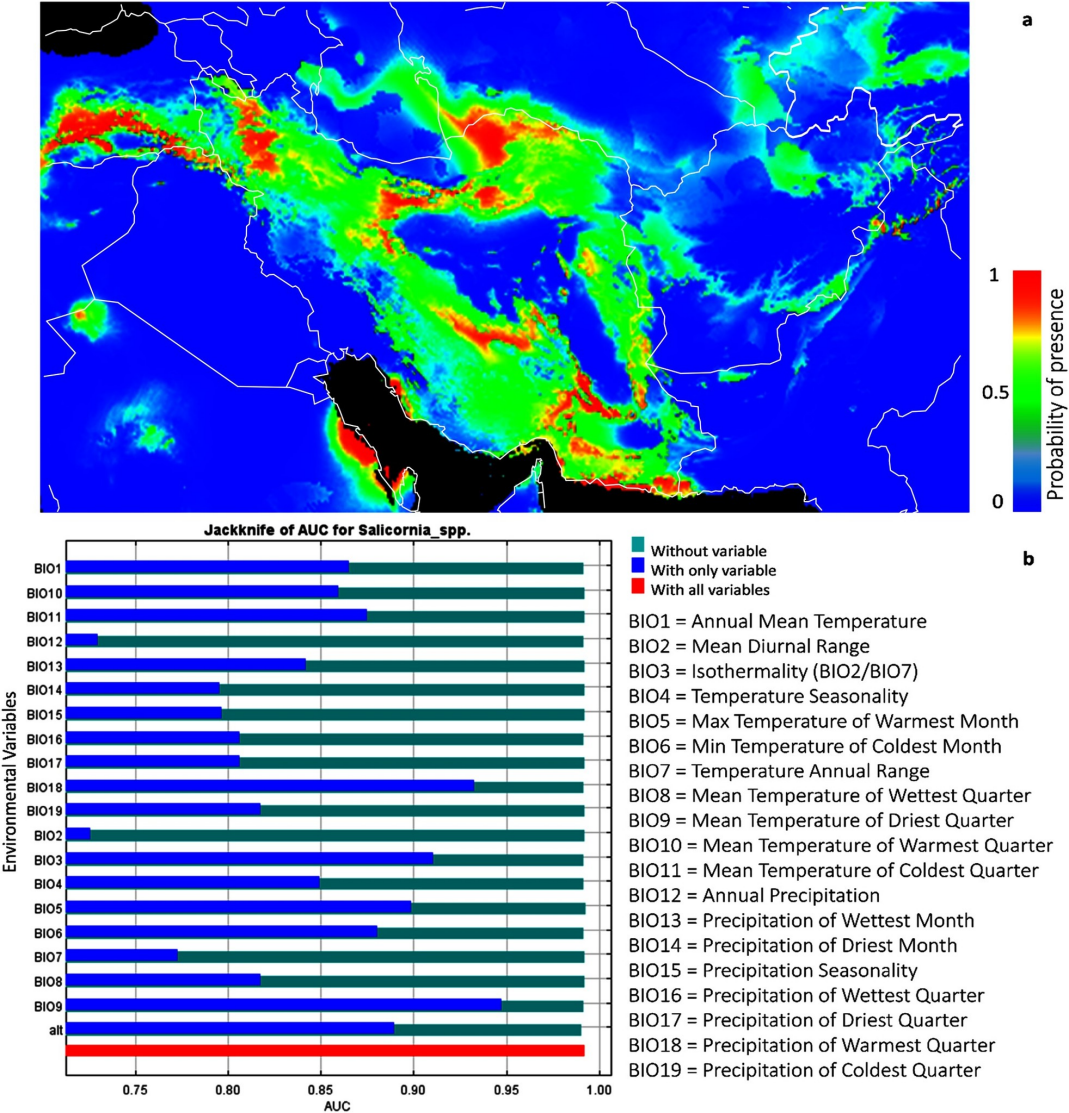
Geographical distribution of *Salicornia* in Iran (a); the probability of presence increase from blue to red, (b) the effect of climates parameters on *Salicornia* distribution in Iran. The map was created using free open source MAXENT software version 3.4.1 (https://biodiversityinformatics.amnh.org/open_source/maxent/).

This information is commonly used for spatial diversity and distribution analysis. Biodiversity of plants is studied at three levels comprising of species-level and genetic level in a population (ecosystem). Here, *Salicornia* was studied at the species level or alpha diversity. In this case, the species level was observed in species diversity and evaluated in being absent or present conditions in each specific area. Further, the determination of diversity (including species, genotype, or ecotype) in different subunits was one of the most crucial aims in this study. The sub-units were areas that *Salicornia* species were found in a previous study. Consequently, diversity was studied at the alpha level, and the sampling areas were mapped using the observed number for each sample and their distributions (Fig 7). The regions were then classified into five groups based on their distributions.

The area with the highest and the lowest number of samples is depicted in red and dark green, respectively, in Fig 7. For example, if a plant protection program is considered, the areas with the highest alpha diversity (red) are in the top priority.

### Ecological niche modelling of *Salicornia* sp

**Fig 8:**
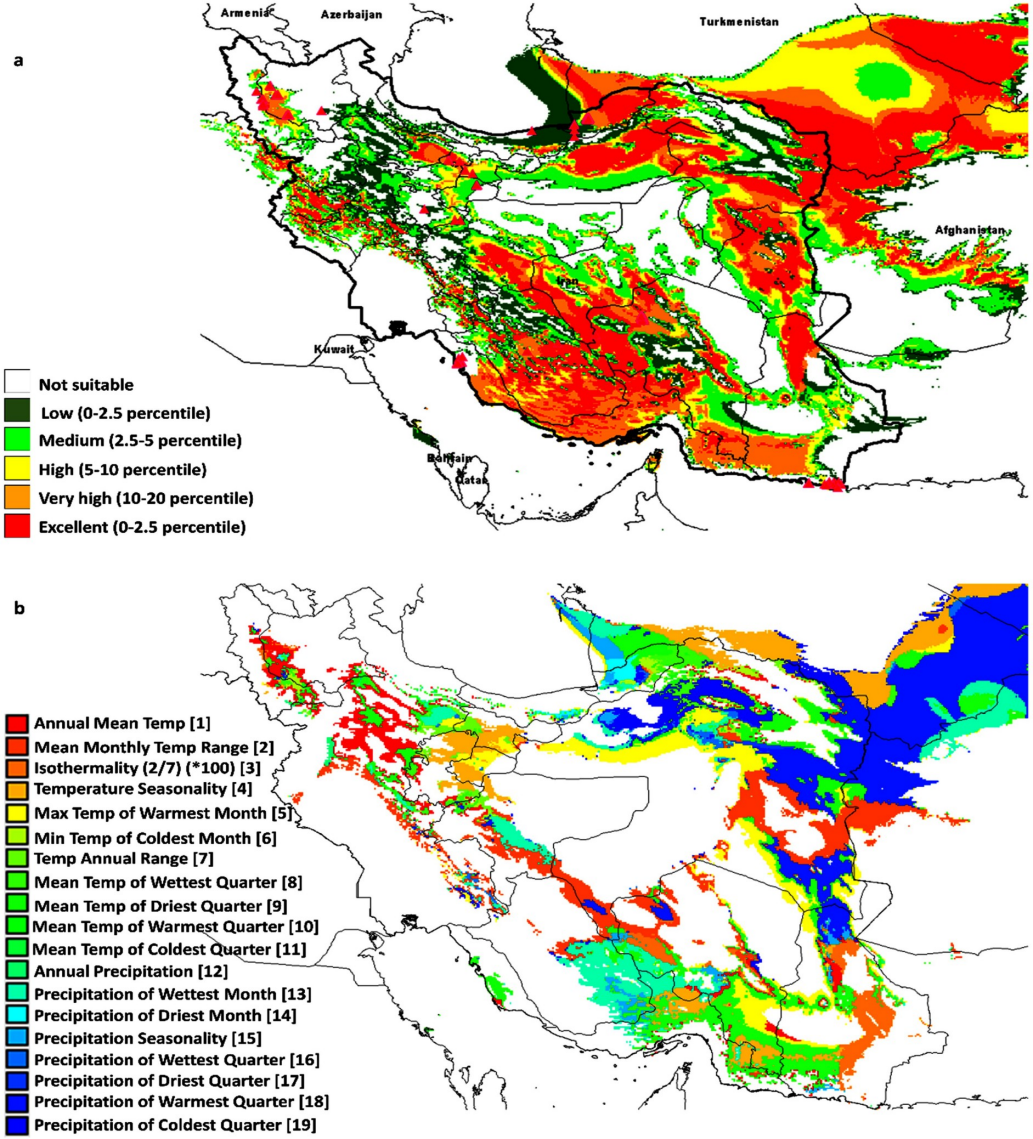
Prediction model of *Salicornia* distribution (a) and limiting factors (b), based on geographical data and cluster analysis of sampled material. Different niches for protection and cultivation of *Salicornia* was shown with red to green color. The most competent areas for natural identification or cultivation of *Salicornia* when optimum water and soil parameter conditions are available shown in red colors while dark green colors show the least potential areas. The map was created using free software DIVA-GIS Version 7.5 (www.diva-gis.org).

We used MAXENT to predict the potential distribution of the species of *Salicornia populations* based on the climatic, environmental variables. In the current study, 19 different climate variables affecting the distribution of species were evaluated [52] (Fig 8 and S2 File). As shown in Fig 8, isothermality (diurnal range mean/temperature annual range) *100, mean temperature of driest quarter, precipitation of warmest quarter, the maximum temperature of the warmest month, altitude, temperature seasonality, mean temperature of coldest quarter, minimum temperature of the coldest month, and annual mean temperature are the most critical variables to the *Salicornia* MAXENT model, based on jackknife test AUC. The average test AUC for each replicate runs were 0.994 with 0.002 standard deviation indicating MAXENT produces highly accurate predictions of AUC value, for all species.

We also studied ecological niches on the conservation and utilization of genetic resources. This concept has been used to determine the priority of areas suitable for the conservation of wild species or the banking of natural genetic resources. Ecological niches are occupied area by a species in natural environmental conditions. Initial niches were also identified in this study. These areas have favorable climatic conditions for *Salicornia* growth and can be used to found these species.

Further, in the field trial condition, the coverage of the primary and ecological niches was confirmed. However, in some primary niches, *Salicornia* samples were not found due to the negative effect of climates or ecological parameters. Further, Geographic Information Systems (GIS) was used to model ecosystem nets based on available environmental data of each collected sample [15]. This model was drawn using DIVA and MAXENT software (Fig 8). The map showed different niches for the protection and cultivation of *Salicornia*. The most competent areas for natural identification or cultivation of *Salicornia* are shown in red colors where optimum water and soil parameters are available. However, the dark green colors show the least potential regions. Together, the model exhibited saline soil, and inefficient land might be used for industrial cultivation of *Salicornia* in Iran. Murray‐smith et al. (2009), previously applied MAXENT modeling and DIVA-GIS analysis to identify priority areas for the conservation of Myrtaceae. Their model showed observed species occurrences and predicted species occurrences and indicated complementarity analysis congruent in identifying areas with the most endemic species.

## Conclusion

We validated genes in the field of DNA barcoding in *Salicornia* plants using *matK*, *rbcL*, *trnH-psbA*, ycf and *ITS2*, identifying species groups. Among the genetic markers tested, *rbcL* had the highest amplification and recoverability rates (98.90%), followed by *trnH*-*psbA* (82.42%), *matK* (80.21%), *ycf* (69.23%) and the rate for *ITS2* was the lowest (65.93%). Data integration showed identification at the level of the species group was higher. This group included *S*. *persica*, *S*. *europea*, *S*. *patula*, *S*. *brachiate*, *S*. *herbacea*, and *S*. *maritime*. Molecular variance analysis and *F*_*st*_ index for plastid and nuclear markers showed genetic differences within the population of the same species is greater than between the populations indicating a high genetic diversity among the genotypes of each population. We also studied ecological niches on conservation and utilization of genetic resources to determine the priority of areas suitable for conservation of wild species or natural genetic resources banking. Our results provided valuable information on the diversity of specific geographical regions, conservation status of existing species, prioritization of conservation areas, and selection of regions for Agro-Ecological, which might be led to the development of industrial agriculture.

## Supporting information

S1 Fig Taxonomic identifications based on the combination of the identity score value. (High identity: X≥95%; Medium identity: 90% ≤ X ≤ 95%; Low identity: X ≤ 90%).
(TIF)

S2 Fig Automatic Barcode Gap Discovery (ABGD) analysis of barcoding markers, (a) Histogram of distances, (b) graph of ranked distances, (c) Automatic partition.
(TIF)

S1 Table DNA barcode primers sequences used in this study. (DOCX)

S1 File The phylogenetic tree using a single fragment of *ITS2*, *matK*, *rbcl*, *trn* and *ycf*. (PDF)

S2 File The primary data used in DIVA and MAXENT software. (PDF)
