# Supplementary figures and images for: Application of DNA barcodes and spatial analysis in conservation genetics and modeling of Iranian Salicornia genetic resources
Source: PLoS One. 2021 Apr 23;16(4):e0241162. doi: 10.1371/journal.pone.0241162 (PMC8064562; doi:10.1371/journal.pone.0241162)

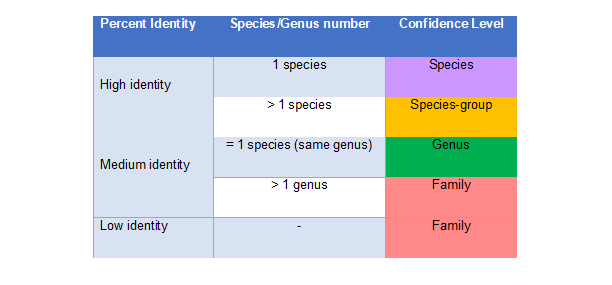

Supplement: S1 Fig — (High identity: X≥95%; Medium identity: 90% ≤ X ≤ 95%; Low identity: X ≤ 90%). (TIF) [file pone.0241162.s001.TIF]

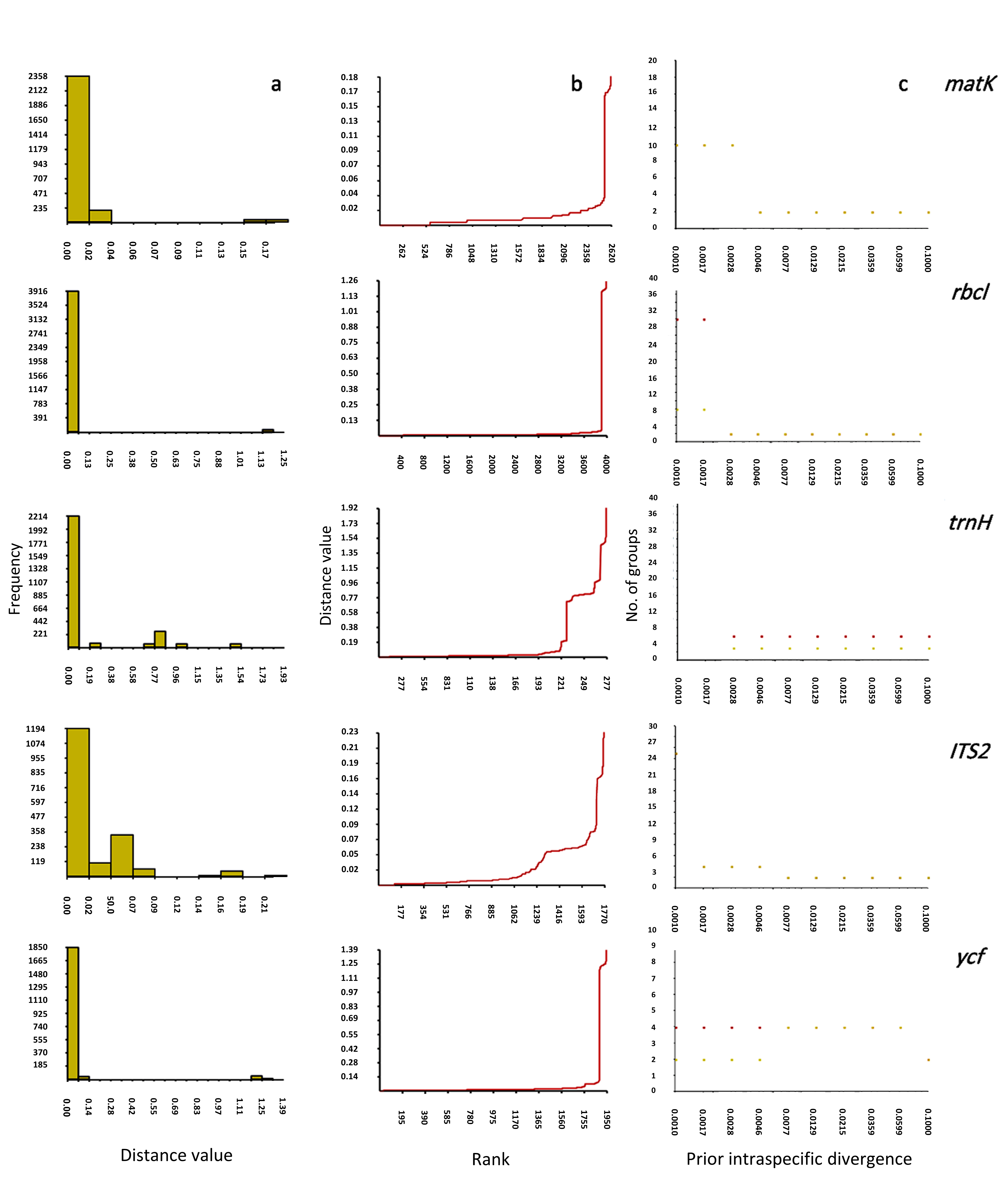

Supplement: S2 Fig — Automatic Barcode Gap Discovery (ABGD) analysis of barcoding markers, (a) Histogram of distances, (b) graph of ranked distances, (c) Automatic partition. (TIF) [file pone.0241162.s002.tif]
