# Supplementary material for: Application of DNA barcodes and spatial analysis in conservation genetics and modeling of Iranian Salicornia genetic resources
Source: PLoS One. 2021 Apr 23;16(4):e0241162. doi: 10.1371/journal.pone.0241162 (PMC8064562; doi:10.1371/journal.pone.0241162)
Supplement: S1 Table — (DOCX) [file pone.0241162.s003.docx]

**Application of DNA barcodes and spatial analysis in conservation genetics and modeling of Iranian Salicornia genetic resources**

Mehrshad Zeinalabedini^1*^, Nayer Azam Khoshkholgh Sima^2*^, Mohammad Reza Ghaffari^1^, Ali Ebadi^2^ and Maryam Farsi^1^

Table S1. DNA barcode primers sequences used in this study.

| Primers | Sequence |
| --- | --- |
| *matK* _For (390) | 5ꞌ-TGTAGCACAGGAAAGTCGAAGT-3ꞌ |
| *matK* _Rev (1326) | 5ꞌ-CGATCTATTCATTCAATATTTC-3ꞌ |
| *rbcLa* _For | 5ꞌ-ATGTCACCAACAAACAGAGACTAAAGC-3ꞌ |
| *rbcLa* _Rev | 5ꞌ-GTAAAATCAAGTCCACCRGC-3ꞌ |
| *trnH-psbA* _For | 5ꞌ-ACTGCCTTGATCCACTTGGC-3ꞌ |
| *trnH-psbA* _Rev | 5ꞌ-CGAAGCACCATCTACAAATGG-3ꞌ |
| *ITS2*_For | 5ꞌ-CGTAACAAGGTTTCCGTAGGTGAACC-3ꞌ |
| *ITS2*_Rev | 5ꞌ-TTATTGATATGCTTAAACTCAGCGGG-3ꞌ |
| *Ycf 1b*_For | 5ꞌ-TCTGGACGAAAATCAGATTGTTGTGAAT-3ꞌ |
| *Ycf 1b*_Rev | 5ꞌ-ATACATGTTCAAAGTGATGGAAAA-3ꞌ |
