# Supplementary material for: Application of DNA barcodes and spatial analysis in conservation genetics and modeling of Iranian Salicornia genetic resources
Source: PLoS One. 2021 Apr 23;16(4):e0241162. doi: 10.1371/journal.pone.0241162 (PMC8064562; doi:10.1371/journal.pone.0241162)
Supplement: S1 File — (PDF) [file pone.0241162.s004.pdf]

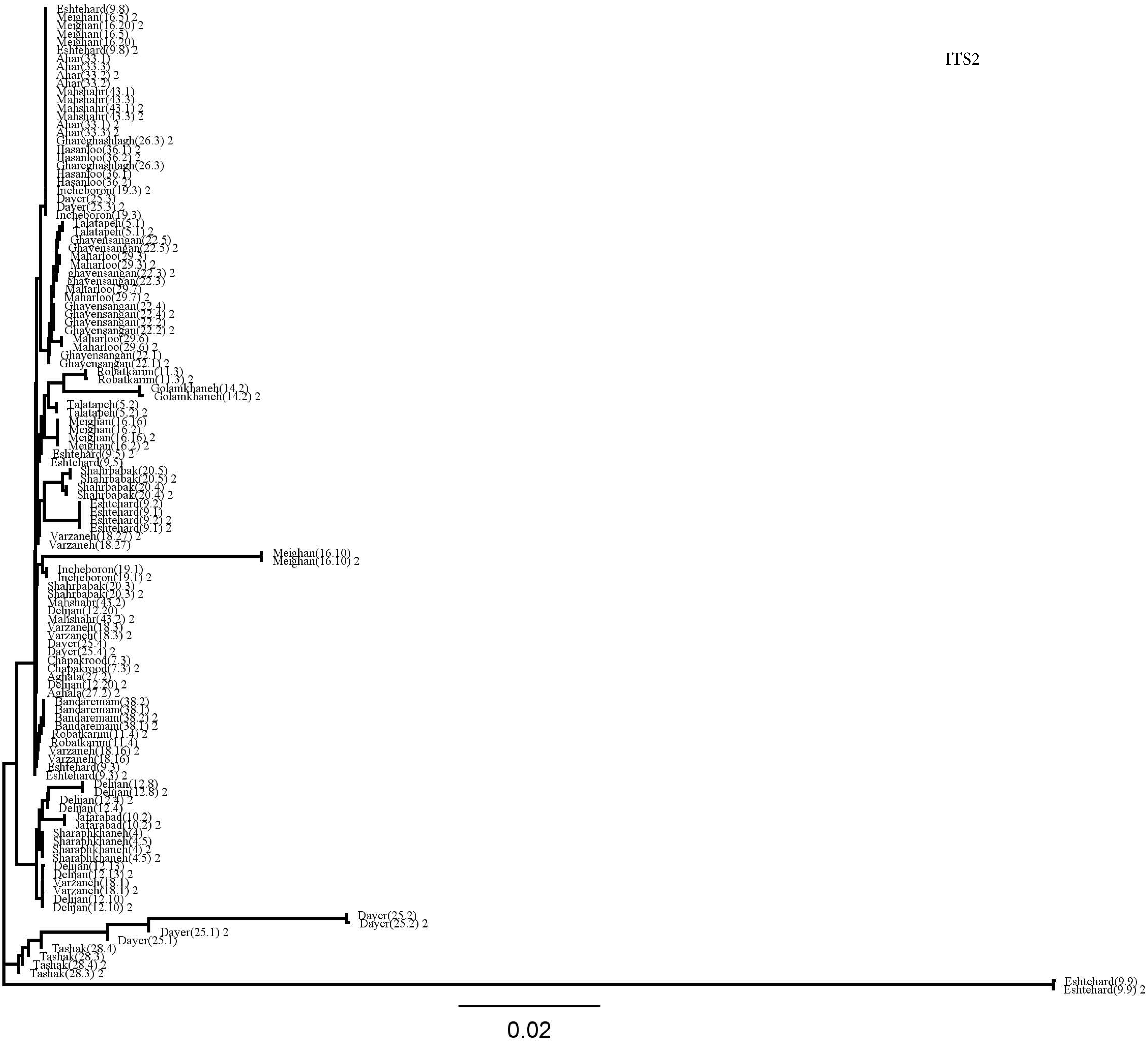
ITS2
Eshtehard(9.8)
Meighan(16.5) 2
Meighan(16.20) 2
Meighan(16.5)
Meighan(16.20)
Eshtehard(9.8) 2
Ahar(33.1)
Ahar(33.3)
Ahar(33.2) 2
Ahar(33.2)
Mahshahr(43.1)
Mahshahr(43.3)
Mahshahr(43.1) 2
Mahshahr(43.3) 2
Ahar(33.1) 2
Ahar(33.3) 2
Ghareghashlagh(26.3) 2
Hasanloo(36.1) 2
Hasanloo(36.2) 2
Ghareghashlagh(26.3)
Hasanloo(36.1)
Hasanloo(36.2)
Incheboron(19.3) 2
Dayer(25.3)
Dayer(25.3) 2
Incheboron(19.3)
Talatapeh(5.1)
Talatapeh(5.1) 2
Ghayensangan(22.5)
Ghayensangan(22.5) 2
Maharloo(29.3)
Maharloo(29.3) 2
ghayensangan(22.3) 2
ghayensangan(22.3)
Maharloo(29.7)
Maharloo(29.7) 2
Ghayensangan(22.4)
Ghayensangan(22.4) 2
Ghayensangan(22.2)
Ghayensangan(22.2) 2
Maharloo(29.6)
Maharloo(29.6) 2
Ghayensangan(22.1)
Ghayensangan(22.1) 2
Robatkarim(11.3)
Robatkarim(11.3) 2
Golamkhaneh(14.2)
Golamkhaneh(14.2) 2
Talatapeh(5.2)
Talatapeh(5.2) 2
Meighan(16.16)
Meighan(16.2)
Meighan(16.16) 2
Meighan(16.2) 2
Eshtehard(9.5) 2
Eshtehard(9.5)
Shahrbabak(20.5)
Shahrbabak(20.5) 2
Shahrbabak(20.4)
Shahrbabak(20.4) 2
Eshtehard(9.2)
Eshtehard(9.1)
Eshtehard(9.2) 2
Eshtehard(9.1) 2
Varzaneh(18.27) 2
Varzaneh(18.27)
Meighan(16.10)
Meighan(16.10) 2
Incheboron(19.1)
Incheboron(19.1) 2
Shahrbabak(20.3)
Shahrbabak(20.3) 2
Mahshahr(43.2)
Delijan(12.20)
Mahshahr(43.2) 2
Varzaneh(18.3)
Varzaneh(18.3) 2
Dayer(25.4)
Dayer(25.4) 2
Chapakrood(7.3)
Chapakrood(7.3) 2
Aghala(27.2)
Delijan(12.20) 2
Aghala(27.2) 2
Bandaremam(38.2)
Bandaremam(38.1)
Bandaremam(38.2) 2
Bandaremam(38.1) 2
Robatkarim(11.4) 2
Robatkarim(11.4)
Varzaneh(18.16) 2
Varzaneh(18.16)
Eshtehard(9.3)
Eshtehard(9.3) 2
Delijan(12.8)
Delijan(12.8) 2
Delijan(12.4) 2
Delijan(12.4)
Jafarabad(10.2)
Jafarabad(10.2) 2
Sharaphkhaneh(4)
Sharaphkhaneh(4.5)
Sharaphkhaneh(4) 2
Sharaphkhaneh(4.5) 2
Delijan(12.13)
Delijan(12.13) 2
Varzaneh(18.1)
Varzaneh(18.1) 2
Delijan(12.10)
Delijan(12.10) 2
Dayer(25.2)
Dayer(25.2) 2
Dayer(25.1) 2
Dayer(25.1)
Tashak(28.4)
Tashak(28.3)
Tashak(28.4) 2
Tashak(28.3) 2
Eshtehard(9.9)
Eshtehard(9.9) 2
0.02

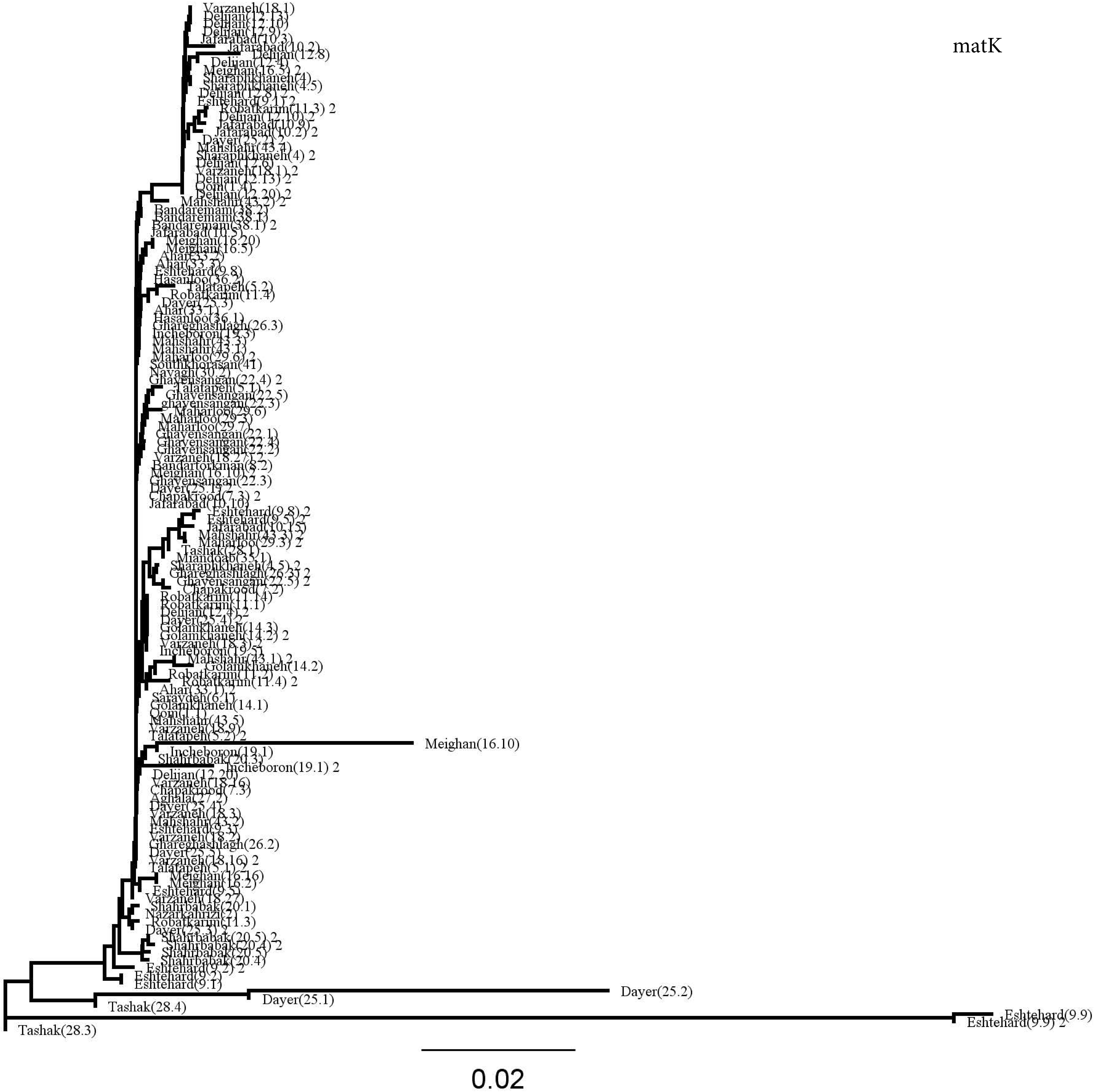

matK
Varzaneh(18.1)
Jafarabad(10.2)
Delijan(12.8)
Delijan(12.4)
Sharaphkhaneh(4.5)
Robatkarim(11.3) 2
Jafarabad(10.2) 2
Sharaphkhaneh(4) 2
Bandaremam(38.1) 2
Jafarabad(10.5)
Meighan(16.20)
Meighan(16.5)
Eshtehard(9.8)
Talatapeh(5.2)
Robatkarim(11.4)
Ghareghashlagh(26.3)
Southkhorasan(41)
Ghayensangan(22.4) 2
Ghayensangan(22.5)
Ghayensangan(22.1)
Bandartorkman(8.2)
Ghayensangan(22.3)
Chapakrood(7.3) 2
Jafarabad(10.10)
Eshtehard(9.8) 2
Jafarabad(10.15)
Maharloo(29.3) 2
Miandoab(35.1)
Sharaphkhaneh(4.5) 2
Ghayensangan(22.5) 2
Chapakrood(7.2)
Golamkhaneh(14.3)
Golamkhaneh(14.2) 2
Mahshahr(43.1) 2
Golamkhaneh(14.2)
Robatkarim(11.2)
Robatkarim(11.4) 2
Golamkhaneh(14.1)
Mahshahr(43.5)
Talatapeh(5.2) 2
Meighan(16.10)
Incheboron(19.1)
Incheboron(19.1) 2
Chapakrood(7.3)
Ghareghashlagh(26.2)
Varzaneh(18.16) 2
Meighan(16.16)
Meighan(16.2)
Shahrbabak(20.1)
Robatkarim(11.3)
Shahrbabak(20.5) 2
Shahrbabak(20.4) 2
Shahrbabak(20.4)
Eshtehard(9.2) 2
Eshtehard(9.2)
Eshtehard(9.1)
Dayer(25.2)
Dayer(25.1)
Tashak(28.4)
Eshtehard(9.9)
Eshtehard(9.9) 2
Tashak(28.3)
0.02

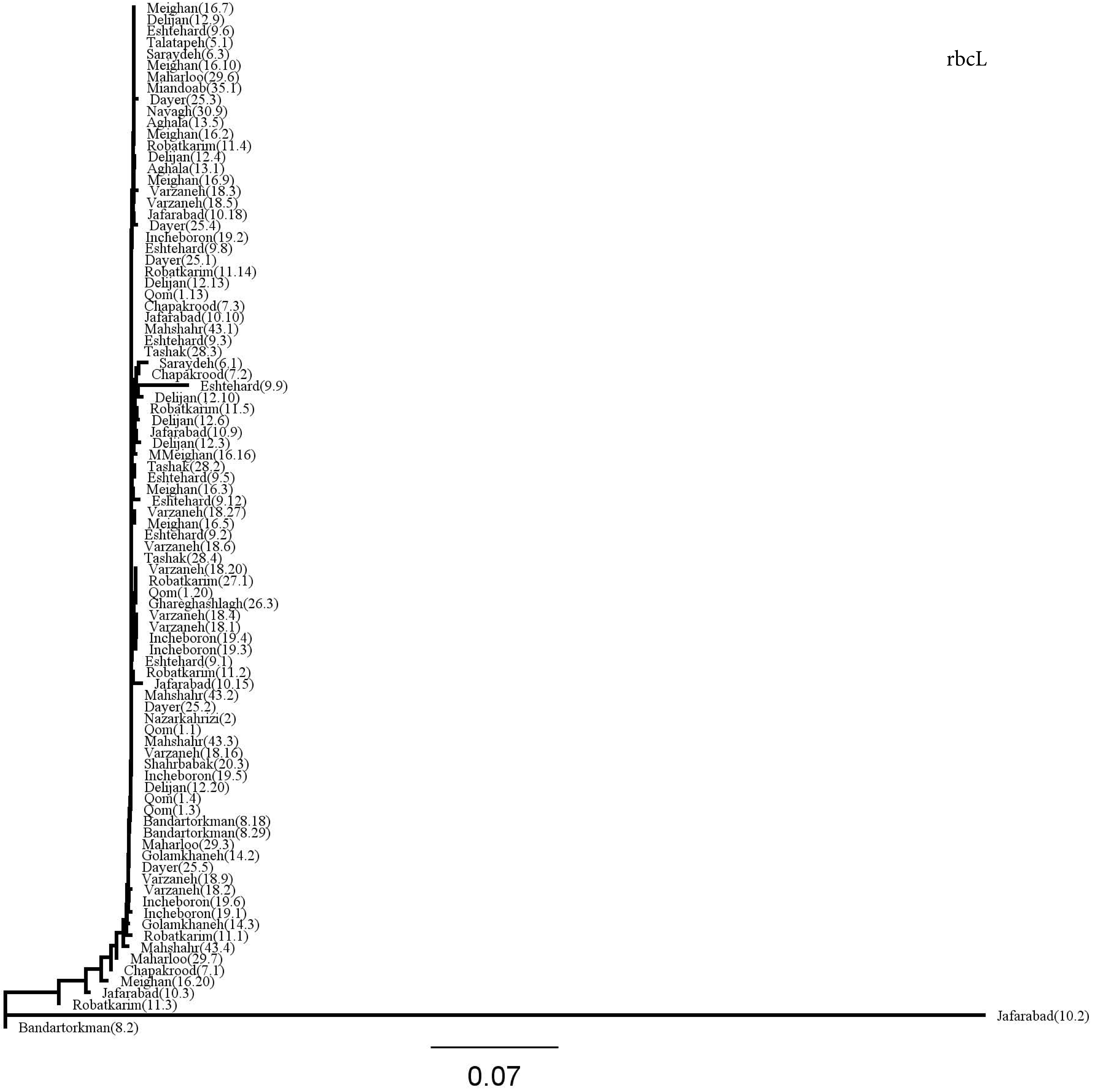
rbcL
Meighan(16.7)
Delijan(12.9)
Eshtehard(9.6)
Talatapeh(5.1)
Saraydeh(6.3)
Meighan(16.10)
Maharloo(29.6)
Miandoab(35.1)
Dayer(25.3)
Navagh(30.9)
Aghala(13.5)
Meighan(16.2)
Robatkarim(11.4)
Delijan(12.4)
Aghala(13.1)
Meighan(16.9)
Varzaneh(18.3)
Varzaneh(18.5)
Jafarabad(10.18)
Dayer(25.4)
Incheboron(19.2)
Eshtehard(9.8)
Dayer(25.1)
Robatkarim(11.14)
Delijan(12.13)
Qom(1.13)
Chapakrood(7.3)
Jafarabad(10.10)
Mahshahr(43.1)
Eshtehard(9.3)
Tashak(28.3)
Saraydeh(6.1)
Chapakrood(7.2)
Eshtehard(9.9)
Delijan(12.10)
Robatkarim(11.5)
Delijan(12.6)
Jafarabad(10.9)
Delijan(12.3)
MMeighan(16.16)
Tashak(28.2)
Eshtehard(9.5)
Meighan(16.3)
Eshtehard(9.12)
Varzaneh(18.27)
Meighan(16.5)
Eshtehard(9.2)
Varzaneh(18.6)
Tashak(28.4)
Varzaneh(18.20)
Robatkarim(27.1)
Qom(1.20)
Ghareghashlagh(26.3)
Varzaneh(18.4)
Varzaneh(18.1)
Incheboron(19.4)
Incheboron(19.3)
Eshtehard(9.1)
Robatkarim(11.2)
Jafarabad(10.15)
Mahshahr(43.2)
Dayer(25.2)
Nazarkahrizi(2)
Qom(1.1)
Mahshahr(43.3)
Varzaneh(18.16)
Shahrbabak(20.3)
Incheboron(19.5)
Delijan(12.20)
Qom(1.4)
Qom(1.3)
Bandartorkman(8.18)
Bandartorkman(8.29)
Maharloo(29.3)
Golamkhaneh(14.2)
Dayer(25.5)
Varzaneh(18.9)
Varzaneh(18.2)
Incheboron(19.6)
Incheboron(19.1)
Golamkhaneh(14.3)
Robatkarim(11.1)
Mahshahr(43.4)
Maharloo(29.7)
Chapakrood(7.1)
Meighan(16.20)
Jafarabad(10.3)
Robatkarim(11.3)
Jafarabad(10.2)
Bandartorkman(8.2)
0.07

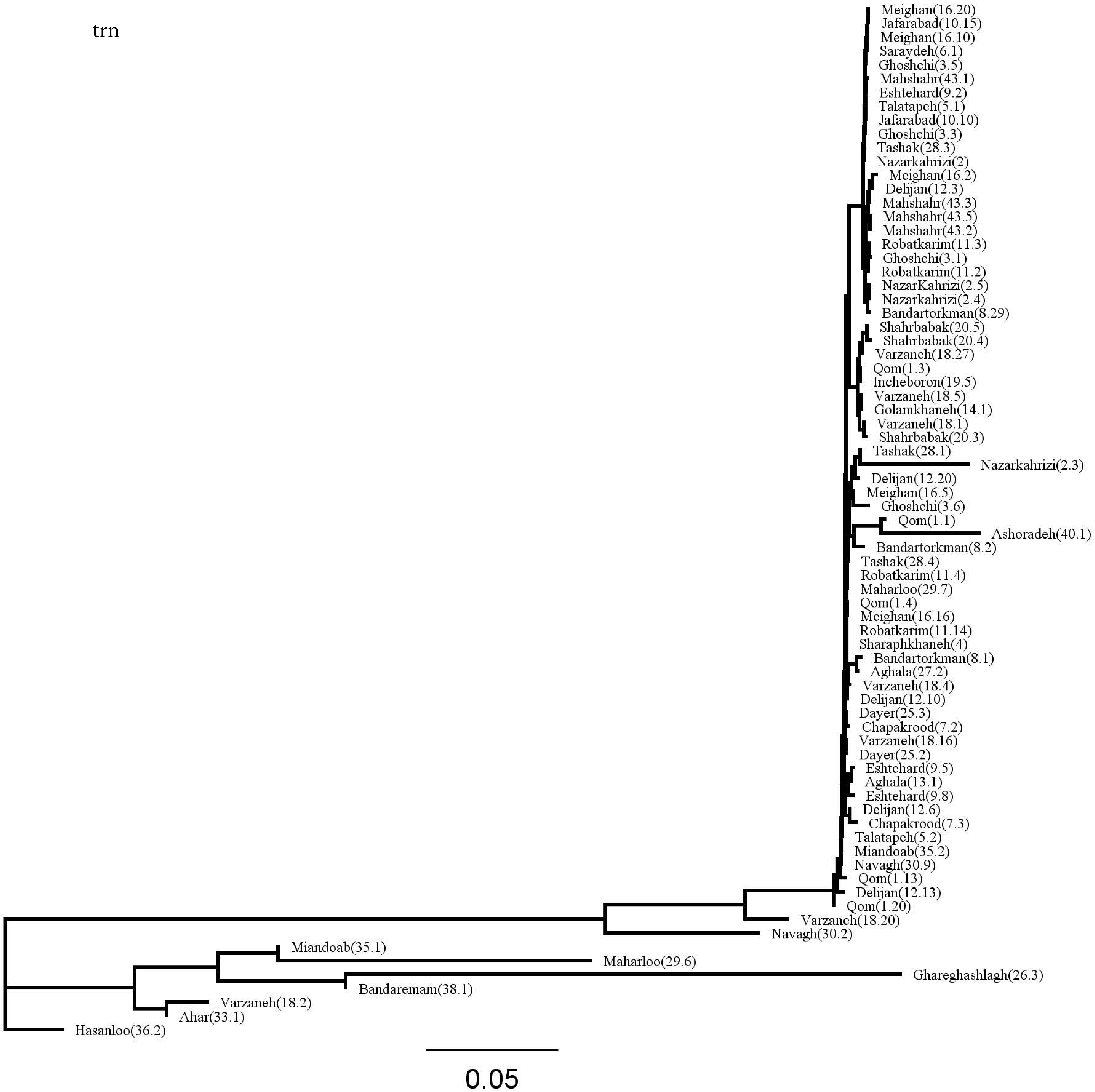
trn
Meighan(16.20)
Jafarabad(10.15)
Meighan(16.10)
Saraydeh(6.1)
Ghoshchi(3.5)
Mahshahr(43.1)
Eshtehard(9.2)
Talatapeh(5.1)
Jafarabad(10.10)
Ghoshchi(3.3)
Tashak(28.3)
Nazarkahrizi(2)
Meighan(16.2)
Delijan(12.3)
Mahshahr(43.3)
Mahshahr(43.5)
Mahshahr(43.2)
Robatkarim(11.3)
Ghoshchi(3.1)
Robatkarim(11.2)
NazarKahrizi(2.5)
Nazarkahrizi(2.4)
Bandartorkman(8.29)
Shahrbabak(20.5)
Shahrbabak(20.4)
Varzaneh(18.27)
Qom(1.3)
Incheboron(19.5)
Varzaneh(18.5)
Golamkhaneh(14.1)
Varzaneh(18.1)
Shahrbabak(20.3)
Tashak(28.1)
Nazarkahrizi(2.3)
Delijan(12.20)
Meighan(16.5)
Ghoshchi(3.6)
Qom(1.1)
Ashoradeh(40.1)
Bandartorkman(8.2)
Tashak(28.4)
Robatkarim(11.4)
Maharloo(29.7)
Qom(1.4)
Meighan(16.16)
Robatkarim(11.14)
Sharaphkhaneh(4)
Bandartorkman(8.1)
Aghala(27.2)
Varzaneh(18.4)
Delijan(12.10)
Dayer(25.3)
Chapakrood(7.2)
Varzaneh(18.16)
Dayer(25.2)
Eshtehard(9.5)
Aghala(13.1)
Eshtehard(9.8)
Delijan(12.6)
Chapakrood(7.3)
Talatapeh(5.2)
Miandoab(35.2)
Navagh(30.9)
Qom(1.13)
Delijan(12.13)
Qom(1.20)
Varzaneh(18.20)
Navagh(30.2)
Miandoab(35.1)
Maharloo(29.6)
Ghareghashlagh(26.3)
Bandaremam(38.1)
Varzaneh(18.2)
Ahar(33.1)
Hasanloo(36.2)
0.05

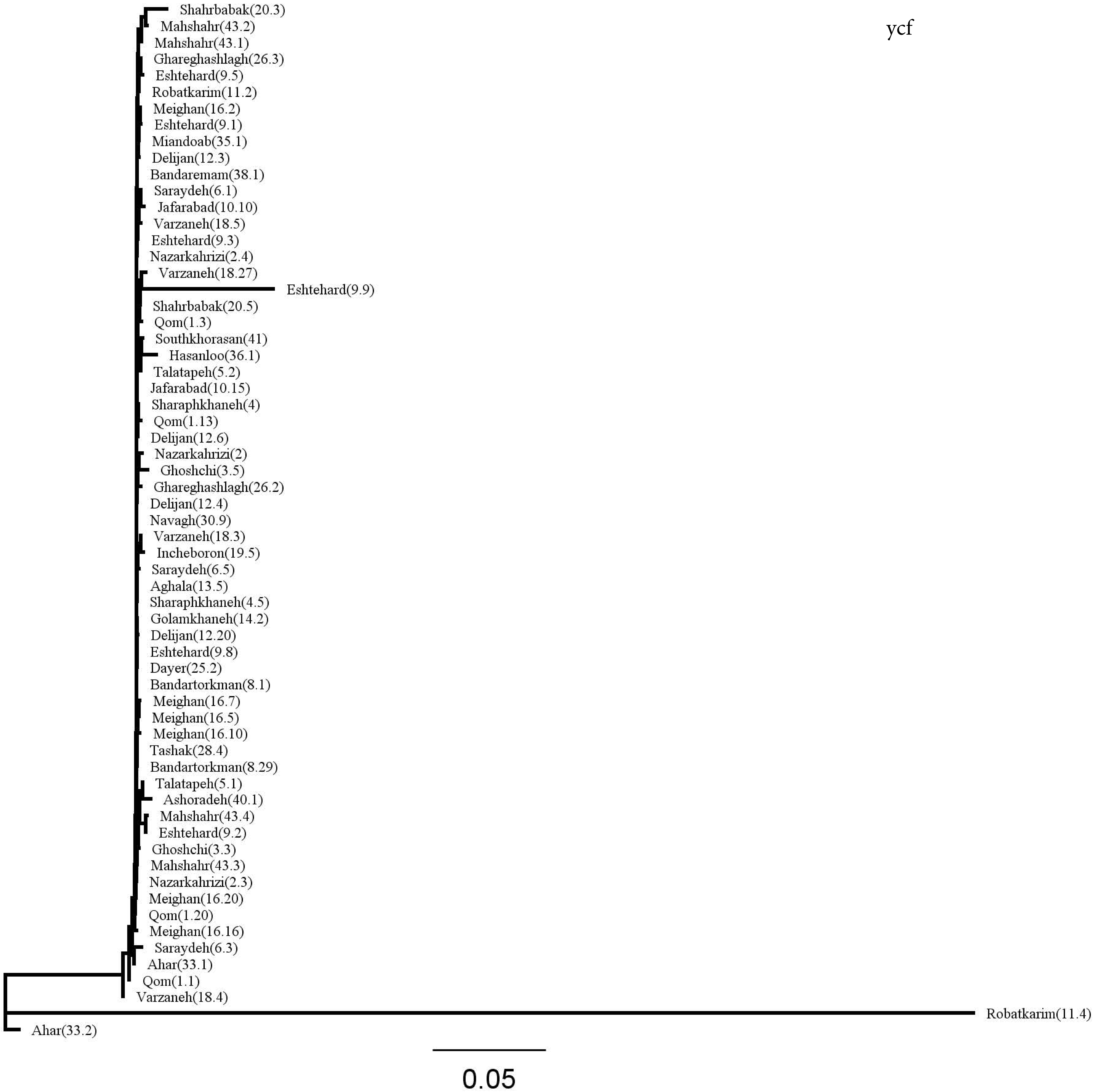
ycf
Shahrbabak(20.3)
Mahshahr(43.2)
Mahshahr(43.1)
Ghareghashlagh(26.3)
Eshtehard(9.5)
Robatkarim(11.2)
Meighan(16.2)
Eshtehard(9.1)
Miandoab(35.1)
Delijan(12.3)
Bandaremam(38.1)
Saraydeh(6.1)
Jafarabad(10.10)
Varzaneh(18.5)
Eshtehard(9.3)
Nazarkahrizi(2.4)
Varzaneh(18.27)
Eshtehard(9.9)
Shahrbabak(20.5)
Qom(1.3)
Southkhorasan(41)
Hasanloo(36.1)
Talatapeh(5.2)
Jafarabad(10.15)
Sharaphkhaneh(4)
Qom(1.13)
Delijan(12.6)
Nazarkahrizi(2)
Ghoshchi(3.5)
Ghareghashlagh(26.2)
Delijan(12.4)
Navagh(30.9)
Varzaneh(18.3)
Incheboron(19.5)
Saraydeh(6.5)
Aghala(13.5)
Sharaphkhaneh(4.5)
Golamkhaneh(14.2)
Delijan(12.20)
Eshtehard(9.8)
Dayer(25.2)
Bandartorkman(8.1)
Meighan(16.7)
Meighan(16.5)
Meighan(16.10)
Tashak(28.4)
Bandartorkman(8.29)
Talatapeh(5.1)
Ashoradeh(40.1)
Mahshahr(43.4)
Eshtehard(9.2)
Ghoshchi(3.3)
Mahshahr(43.3)
Nazarkahrizi(2.3)
Meighan(16.20)
Qom(1.20)
Meighan(16.16)
Saraydeh(6.3)
Ahar(33.1)
Qom(1.1)
Varzaneh(18.4)
Robatkarim(11.4)
Ahar(33.2)
0.05
